# Using Machine Learning Methods and Structural Alerts for Prediction of Mitochondrial Toxicity

**DOI:** 10.1002/minf.202000005

**Published:** 2020-03-23

**Authors:** Jennifer Hemmerich, Florentina Troger, Barbara Füzi, Gerhard F.Ecker

**Affiliations:** ^1^ University of Vienna Department of Pharmaceutical Chemistry Althanstr. 14 1090 Vienna Austria

**Keywords:** Toxicology, Structure-activity relationships, machine learning, mitochondrial toxicity, structural alerts

## Abstract

Over the last few years more and more organ and idiosyncratic toxicities were linked to mitochondrial toxicity. Despite well‐established assays, such as the seahorse and Glucose/Galactose assay, an *in silico* approach to mitochondrial toxicity is still feasible, particularly when it comes to the assessment of large compound libraries. Therefore, *in silico* approaches could be very beneficial to indicate hazards early in the drug development pipeline. By combining multiple endpoints, we derived the largest so far published dataset on mitochondrial toxicity. A thorough data analysis shows that molecules causing mitochondrial toxicity can be distinguished by physicochemical properties. Finally, the combination of machine learning and structural alerts highlights the suitability for *in silico* risk assessment of mitochondrial toxicity.

## Graphical abstract

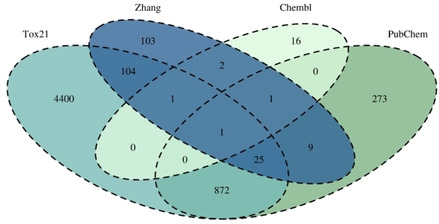

## 1 Introduction

Safety and risk assessment is gaining increasing importance in drug discovery and development. Many drugs which are withdrawn from the market cause idiosyncratic toxicities such as drug induced liver injury, or cardiotoxicity or nephrotoxicity, amongst many others. Two prominent examples are the antidiabetic drug troglitazone and the cholesterol lowering compound cerivastatin. Troglitazone has been withdrawn from the market due to severe liver injury and cerivastatin due to rhabdomyolysis. Later on, the severe adverse effects were linked to mitochondrial toxicity^1^, ^2^ In 2007 Dykens and Will raised the awareness of the commonality of drug induced mitochondrial toxicities.^3^ Since then, the interest in mitochondrial toxicity has increased, as it could not only be linked to acute (idiosyncratic) toxicities (e. g.^4^, ^5^, ^6^) but also to long‐term toxicity such as the induction of Parkinson's disease.^7^, ^8^ Therefore, mitochondrial toxicity is an important endpoint for drug safety. However, the assessment of this toxicity endpoint is not trivial. *In vivo* studies might not show the effects due to poor response of the lab animals or the rare occurrence of idiosyncratic effects. Reasons suggested by Will and Dykes are (i) the animals being young, with strong mitochondrial reserves, and (ii) the genetic diversity within the lab animals is not given.^3^ In addition, *in vivo* mitochondrial toxicity manifests as organ toxicity and the cause thereof cannot be easily determined. *In vitro* studies are therefore more suitable but are either not feasible for high throughput screening, or might not extrapolate very well to the *in vivo* situation^9^ Thus, *in silico* approaches might help in estimating the emanating hazard from such substances. The advantage is that they can be easily conducted and do not need any compound availability. A disadvantage, however, is the lack of suitable, large datasets which might serve as basis for predictive machine learning models. The two biggest published data sets for mitochondrial toxicity are the Zhang data set^10^ (246 compounds) and the Tox21 dataset^11^ (5403 compounds). The Tox21 dataset originates from the Tox21 challenge where the mitochondrial membrane potential was assessed in a high throughput screening assay.^11^ First efforts to characterize mitochondrial toxicity and to derive modes of action for certain structural alerts, were done by Nelms and co‐workers (based on the Zhang dataset) and by Naven and co‐workers (based on an unpublished dataset).^12^, ^13^ In this paper we present the – to our knowledge – biggest dataset for mitochondrial toxicity. After an exhaustive analysis of physicochemical properties of its compounds we show that our dataset can be used to train machine learning models. Furthermore, structural alerts were derived from this data. A retrospective analysis of DrugBank highlights that the combination of machine learning and structural alerts is able to identify mitotoxic drugs. Using structural alerts, we could further derive a mode of action based on the alert.

## 2 Material and Methods

### 2.1 Data Collection

The data was collected by accessing several public platforms. A manual assay search with the keywords “mitochondria”, “mitochondria potential” and “mitochondria complex” in the ChEMBL release 22 database^14^ was performed for binding assays. The aim was to retrieve compounds tested in connection to mitochondrial function, binding and inhibition. The retrieved compounds were separated into “positives” and “negatives”. For the positive compounds the cut‐off was set at an activity value of pChEMBL≥5. The pChEMBL value is available in the ChEMBL database and allows the comparison between different activity types. It is defined as: ‐log (molar IC50, XC50, EC50, AC50, Ki, Kd or Potency). This assay search yielded 21 compounds.

The main source of information was a confirmatory assay from the Tox21 dataset for mitochondrial membrane potential disruption (AID=720637). The assay was retrieved from PubChem and yielded 5403 compounds. Like ChEMBL, PubChem was mined for additional data. This resulted in additional 1181 compounds. For the Tox21 data, as well as for the additional PubChem data, the activity labels as assigned by PubChem were used.

**Figure 1:**
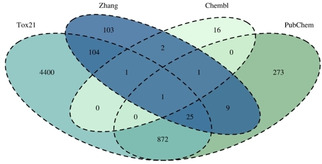
Venn Diagram of the overlap of the merged datasets.

Finally 246 tested drugs and drug like molecules from the publication by Zhang *et al*. were added to the collected data.^10^ The labels “active” and “inactive” were adapted from the publication. In the publication compounds which have been reported to induce mitochondrial toxicity were collected from different literature resources and labelled as “actives”. The “inactives” from the publication are FDA‐approved, common and safe oral drugs with known mode of action which mechanisms are not associated with the mechanisms of mitochondrial toxicity. For an overview of the overlap of the retrieved data see Figure 1. For our dataset we changed the label “active” to “positive” and the label “inactive” to “negative.

### 2.2 Data Standardisation

Via the KNIME 3.6.2 graphical analytics platform a data miner workflow was created^15^ in order to standardise the retrieved compounds and to combine the datasets. The downloaded files were read into the platform. In the case of the PubChem Assays, the PubChem Compounds node was used to map the identifiers to molecular structures in sdf format. The drugs from the *Zhang et al. p*ublication, as well as the downloaded compounds from ChEMBL were added to the workflow via two separate Excel reader nodes. Name and ChEMBL identifier mapping were performed via the Chemical Identifier Resolver node, for duplicate filtering again the standardisation procedure described above was applied. Furthermore, all compounds from all datasets were standardised with our in‐house standardiser version 0.1.6. Standardisation consisted of: (i) breaking bonds to group I and group II metals, (ii) neutralizing charges, (iii) application of rules to standardise the representation of functional groups, (iv) neutralize, (v) discard salts and compounds without any carbons, (vi) removing stereochemistry. For standardisation the RDKit python library was used and implemented within a python scripting node in KNIME.^16^ After standardisation InChIKeys were generated to identify duplicate compounds. The workflow can be downloaded from our GitHub page at https://github.com/PharminfoVienna/Chemical‐Structure‐Standardisation). Compounds with ambiguous labels were removed from the dataset. After duplicate removal we obtained a final dataset of 5761 compounds with 824 “positives” (i. e. Mitochondrial toxic compounds) and 4937 “negatives” (i. e. Non‐mitochondrial toxic compounds).

### 2.3 Analysis of Structural Properties

To analyse differences in the physicochemical properties between positive and negative compounds, selected chemical properties were calculated. Calculation of the chemical properties was performed using the RDKit Descriptor Calculation node in KNIME 3.6.2. For analysis 25 interpretable 2D descriptors were chosen (see supplement A Table A). To gain a better understanding of the physicochemical properties percentage bar charts as well as boxplots were created. The plots were then analysed for differences in the trends of positive and negative compounds. The analysis was conducted using R (version 3.4.4). For all plots the packages ggplot2 (version 2.2.1) and gridExtra (version 2.3) were used. Further, a principal component analysis (PCA) was performed in R using the function prcomp from base R and visualized using the packages factoextra (version 1.0.5), FactoMineR (version 1.39), ggplot2 (version 2.2.1) and gridExtra (version 2.3).

### 2.4 Substructure Analysis

A substructure analysis of the composed dataset was performed applying fragment‐based methods such as SARpy (version 1.0)^17^ and the RDKit fragmentation node (version 3.3.1) and MOE fragmentation node (Molecular operating environment, CCG, KNIME nodes version 2.4.0) in KNIME. SARpy uses a recursive algorithm for fragmentation and we applied two different settings. First, positives and negatives were selected as target activity class, second, only the positive labelled compounds were used. The target activity class sets the class of compounds which will be used for the extraction of structural alerts e. g. if it is set to positives, only positive compounds will be fragmented to generate substructures. For both runs the fragment size was set to a minimum of two and a maximum of 18 atoms, and their occurrence was set to a minimum of five. The precision was set to minimize the false negatives. Using the MOE node four different fragmentation algorithms were applied: RECAP, Ringblock, ScaffoldTree, and Ringatoms. RECAP is a method to obtain large fragments which can easily be used in chemical synthesis and therefore cleaves only specific bond types.^18^ Ringblock splits the molecules between non‐ring bonds, and the Ringatoms algorithm cleaves bonds which contain at least one ring atom. However, both spare double bonds and bonds involved in charge separation. The ScaffoldTree algorithm generates fragments at the basis of a hierarchical clustering of chemical scaffolds.^19^ The RDKit molecule fragmenter node creates all possible fragments within the set length. Here the fragment size was set to a minimum path length of two bonds and a maximum of ten.

Further analysis of the obtained fragments was performed in KNIME. First, the occurrence of fragments in the whole dataset was counted applying the RDKit substructure counter node. Second, the threshold regarding the overall occurrence of the obtained fragments was set to nine. Finally, the positive predictive value (PPV) for each fragment was calculated (see section 2.6). The fragments were ranked regarding their PPV, and fragments with a PPV of smaller than 0.6 were disregarded for further analysis due to their low specificity. The remaining fragments were examined manually. Criteria for selecting a fragment as structural alert were (i) chemical integrity and (ii) completeness. For example, fragments which contained only parts of rings, small fragments with less than 4 atoms, ubiquitously occurring substructures (such as benzene) or unspecific carbon chains were not considered useful. After the selection 17 structural alerts were identified.

### 2.5 Machine Learning Models

The machine learning models were trained using three different approaches (for an overview see Table 1).

**Table 1:** Model overview. Overview of the descriptors and parameters used for the development of the machine learning models.

Model	Descriptor	Split	Training	Hyper‐parameter search	Balancing technique
Random Forest	10 RDKit	80/20	CV+ External test	no	no
Gradient Boosting	Atom Pair FP	80/20	Nested CV+ External test	Across model algorithms and inputs	no
Deep learning	RDKit all	80/20	Nested CV+ External test	Network architecture	no

One model was trained using an extensive model selection workflow available in the KNIME 3.6.2 analytics platform on the examples server under *04_Analytics/11_Optimization/08_Model_Optimization_and_Selection*. The workflow trains a gradient boosting model, a random forest, a naive Bayes and a logistic regression using five different types of fingerprints: ECFC6, ECFP6, ECFP4, AtomPair, RDKit. For the model training, the dataset is split into test and training set (20 % and 80 % of the data). To find the optimal hyperparameters, a hyperparameter search is conducted splitting the training dataset further into a training and validation set. In addition to the model specific hyperparameters, the hyperparameter search included the choice of the fingerprint used. From all trained models in the hyperparameter search the best model is kept. The final models for each algorithm are then compared and the best performing model is selected. Subsequently, this model is re‐trained with the original training data and validated on the left‐out test dataset.

The second approach used deep learning. The training procedure was similar to the KNIME workflow but splits the data according to a clustering. The clustering is done with affinity propagation^20^, ^21^ and was done using the implementation from the python library scikit‐learn. After the clusters are determined, the clusters are distributed to the folds randomly. This ensures that molecules in the same cluster will be distributed to the same fold. This reduces the model bias, and increases the generalization of the model.^20^, ^21^ For the hyperparameter selection three out of five folds are used and the fourth fold is used for the inner validation. The best hyperparameter set is chosen based on a cross‐validation of the inner four folds and then the final model is tested on the fifth fold. Therefore, an ensemble of five models is created. The deep learning models were trained using RDKit descriptors as available in KNIME.

The third approach is using a RandomForest as implemented in the scikit‐learn library (version 0.19.0) in python with default parameters. For this model we used a smaller, randomly selected, dataset of 1412 compounds. For this approach the descriptors were chosen based on the data analysis and the PCA. The goal was to include as little correlated descriptors as possible (see supplement A Figure A), with a maximum information gain. This resulted in the following descriptors: “SlogP”, “TPSA”, “NumLipinskiHBA”, “NumLipinskiHBD”, “NumRotatableBonds”, “NumHeavyAtoms”, “NumAromaticRings”, ”NumAliphaticRings“, ”NumSaturatedHeterocycles“, ”NumAliphaticHeterocycles“. This new dataset was split into 5 folds and then subjected to a 5‐fold cross‐validation. In addition, the left‐out samples served as an external test set.

Although all models had their own external test set, the comparison is more straightforward and meaningful with a common external test set. For that reason, we used the combined validation and test set from the Tox21 Challenge with the endpoint “SR‐MMP” (stress response – mitochondrial membrane potential), which allowed us to compare the model performance directly. The datasets were downloaded from the challenge homepage (https://tripod.nih.gov/tox21/challenge/data.jsp) and standardized with the workflow described in section 2.1. Duplicate filtering and joining of the two datasets were also done using InChIKeys.

### 2.6 Model Performance

The model performance was evaluated based on the confusion matrix. Since the dataset was imbalanced, we did not take the accuracy of the models into account. Instead, we used the sensitivity, specificity, their harmonic mean (namely the balanced accuracy), and the positive predictive value (PPV) for evaluation. Those metrics can best assess the predictivity of the models in terms of recognition of the positive and negative class separately. In addition, we always considered the confusion matrix to identify bias towards a class. For the deep learning models, which have a continuous output between 0 and 1, we used a threshold of 0.5 for classification. Hence, all molecules which were predicted as 0.5 or below were classified as negatives, all molecules above were classified as positives.

The metrics were calculated as follows:

$$Sensitivity\;=\frac{True\;positives}{True\;positives+False\;negatives}$$

$$Specificity\;=\frac{True\;negatives}{True\;negatives+False\;positives}$$

$$Balanced\;accuracy\;=\frac{Sensitivity+Specificity}{2}$$

$$PPV\;=\frac{True\;positives}{True\;positives+False\;positives}$$

where the sensitivity reflects the recognition of positives and the specificity the recognition of negatives. In addition, the balanced accuracy evaluates the average performance of the model. The PPV gives notion of the percentage of correctly classified positives. For the evaluation of the structural alerts a false positive denotes a negative compound which was flagged by a structural alert.

### 2.7 Applicability Domain Calculation

The applicability domain was calculated using the local outlier factor as described by Breuning and coworkers.^22^ In brief, the algorithm compares the local densities of the nearest neighbors of a compound to its local density. If this factor is below 1 (i. e. the local density of the compound is greater or equal to its surroundings) a compound is considered in domain otherwise a compound is out of domain. To calculate these factors, we used the LOF algorithm as implemented in the python library scikit‐learn. The parameters we used were 5 nearest neighbours, novelty=True, a contamination of 0.1 and the Euclidean metric. As the input we used minmax scaled descriptors and the first two principal components.

### 2.8 Retrospective Analysis of DrugBank

For the analysis we downloaded DrugBank version 5.1.1 and standardised it according to the procedure described above. The respective descriptors and fingerprints for the models were generated and all compounds were predicted using the machine learning models as well as the structural alerts.

For the analysis a KNIME workflow was developed. The drugs which were predicted as positives by all models, and the compounds which were predicted as positives by the alerts, were analysed and an automated literature search was conducted with the terms: “DrugName+mitochondrial+toxicity”, “DrugName+mitochondria+toxicity”, “DrugName+mitochondria”, “DrugName+toxicity” in PubMed. The DrugName indicates the generic drug name as assigned by DrugBank. The retrieved literature was then manually examined for possible evidence of mitochondrial toxicity.

## 3 Results

### 3.1 Dataset Analysis

The only dataset available for mitochondrial toxicity is the dataset by Zhang and co‐workers.^10^ However, it is known that many different endpoints, such as the inhibition of the mitochondrial membrane potential, can lead to mitochondrial toxicity and ultimately to cell death. Especially a loss in the membrane potential can lead to increased ROS (reactive oxygen species) formation or perturbation of the energy homeostasis, thus resulting in mitochondrial toxicity and cell death.^6^, ^23^ Therefore, we combined multiple datasets using the endpoint mitochondrial membrane potential as well as other assays related to mitochondrial toxicity to generate a large dataset for subsequent analysis. The final dataset was compiled from the Zhang dataset, the Tox21 Assay for mitochondrial membrane potential and a manual assay and compound search in both, ChEMBL and PubChem (see section 2.1 and 2.2 and Figure 1). Finally, we obtained a dataset consisting of 5761 compounds with 824 “positives” (i. e. mitochondrial toxic compounds) and 4937 “negatives” (i. e. non‐mitochondrial toxic compounds).

**Figure 2:**
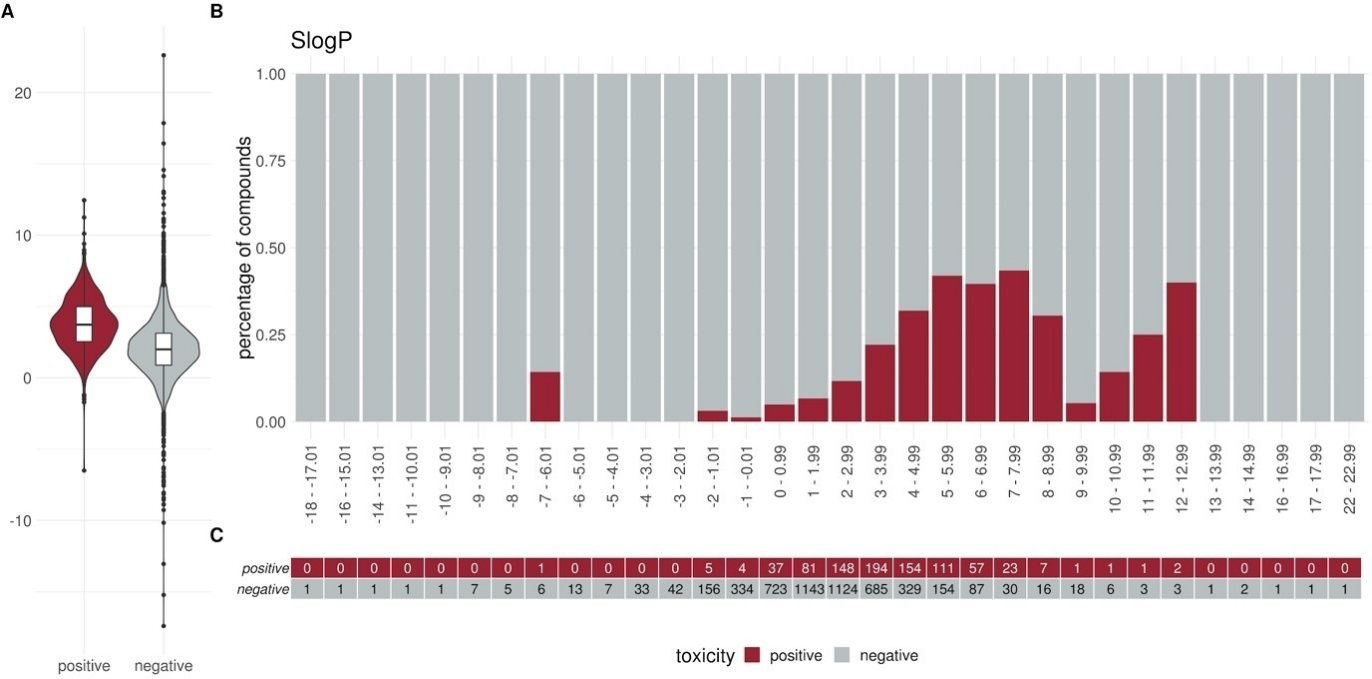
Distribution of the descriptor SlogP (logP) between positives and negatives (A) Box or Violin plots of the distribution of the positives (red) and negatives (grey) compounds in our dataset (without the external test set) (B) Percentage histograms of the dataset. Each bar indicates the percentage of the positives compounds (red) and the percentage of the negatives compounds (grey) for the respective bin. (C) Number of positives (red) and negatives (grey) compounds per bin in the percentage histogram.

To elucidate specific trends in our dataset we plotted different descriptors in a percentage histogram (see Figure 2–Figure 4). The most prominent trend was seen for the descriptor “SlogP”. First, only 1.2 % (10/824) of the positive compounds had a negative SlogP ranging, whereas 12.3 % (607/4931) of the negative compounds showed a negative SlogP. Secondly, in the area of SlogP 4–9, a significant enrichment of positives is observed (>25 %). This indicates that an increased membrane permeability is needed for a compound in order to directly interact with the outer mitochondrial membrane or even cross the outer and interact with the inner mitochondrial membrane. In addition, we observed that the activity seems to be related to the number of aromatic rings (aromatic carbocycles, as well as aromatic cycles in general). The positive compounds follow a normal distribution with a mean of 4–5 for aromatic carbocycles and 3–4 for aromatic rings. This is in line with the findings for the SlogP, since carbocycles are apolar and thus contribute to a high logP. For the molecular weight, the fraction of sp3 hybridized carbons (FractionCSP3) and the surface area (LabuteASA), no strong trends in the histograms are visible. The boxplots in Figure 5 to Figure 7, however, also indicate a shift in the medians between the positives and negatives. This highlights that certain molecular properties seem to be favourable for mitotoxic compounds. In this case the surface area as well as the molecular weight show that most compounds below 250 Da seem to be too small to induce substantial effects. Similarly, most compounds above 600 Da seem to be too large to induce any effect. Since the weight is correlated with the surface area of a molecule the same holds true for compounds with a too small, or too large, surface area. In addition, the fraction of sp3 hybridized carbon is lower in the positive compounds. This is in line with the finding that the activity depends on the number of aromatic rings, indicating a lower number of sp3 hybridized carbons.

**Figure 3:**
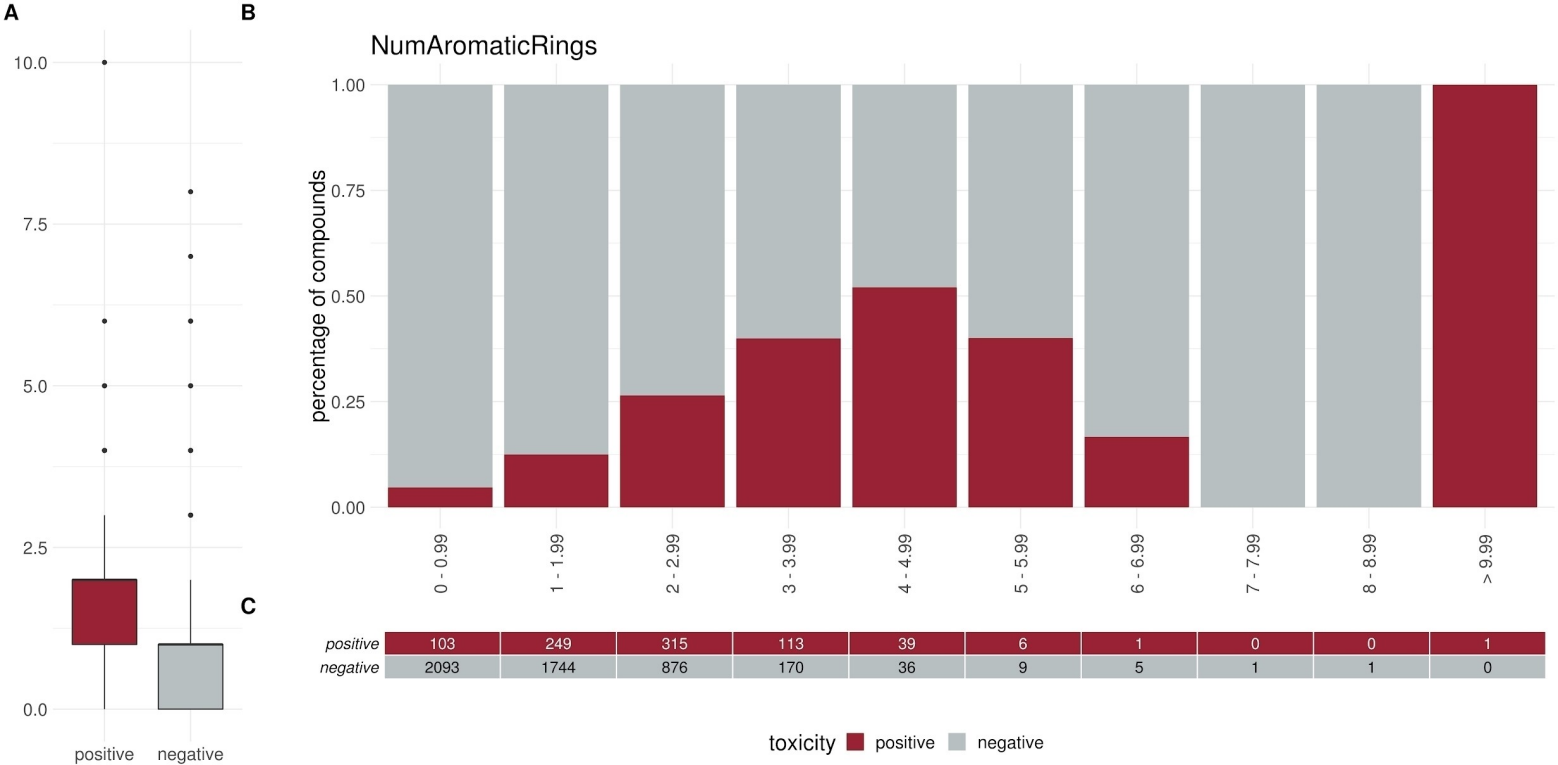
Distribution of the descriptor NumAromaticRings (number of aromatic rings) between positives and negatives. (A) Box or Violin plots of the distribution of the positives (red) and negatives (grey) compounds in our dataset (without the external test set) (B) Percentage histograms of the dataset. Each bar indicates the percentage of the positives compounds (red) and the percentage of the negatives compounds (grey) for the respective bin. (C) Number of positives (red) and negatives (grey) compounds per bin in the percentage histogram.

**Figure 4:**
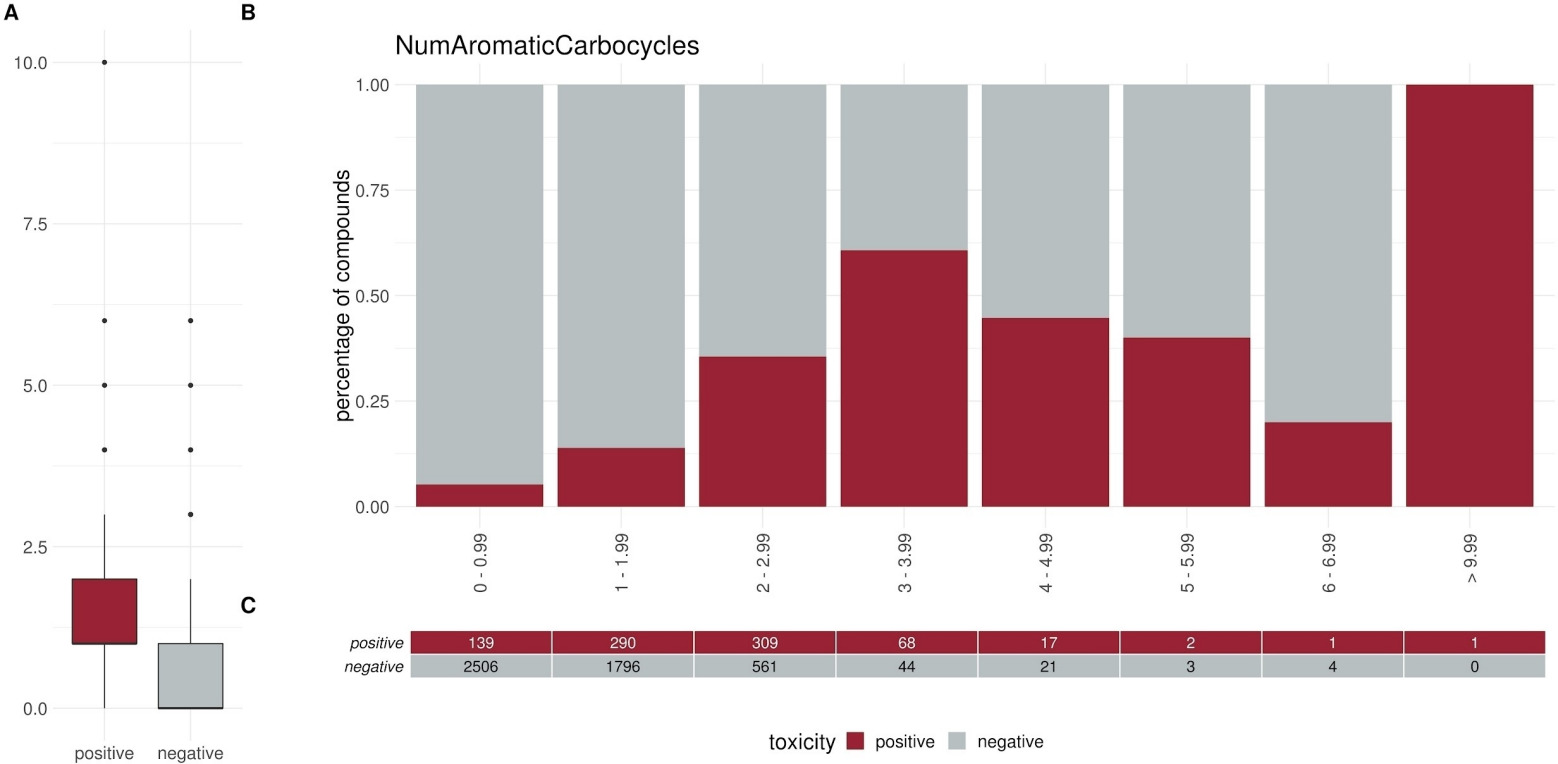
Distribution of the descriptor NumAromaticCarbocycles (number of aromatic carbocycles) between positives and negatives (A) Box or Violin plots of the distribution of the positives (red) and negatives (grey) compounds in our dataset (without the external test set) (B) Percentage histograms of the dataset. Each bar indicates the percentage of the positives compounds (red) and the percentage of the negatives compounds (grey) for the respective bin. (C) Number of positives (red) and negatives (grey) compounds per bin in the percentage histogram

**Figure 5:**
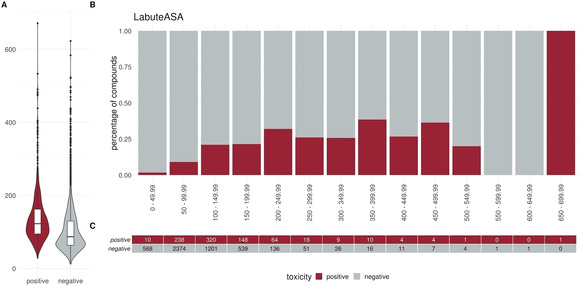
Distribution of the descriptor LabuteASA (surface area) between positives and negatives. (A) Box or Violin plots of the distribution of the positives (red) and negatives (grey) compounds in our dataset (without the external test set) (B) Percentage histograms of the dataset. Each bar indicates the percentage of the positives compounds (red) and the percentage of the negatives compounds (grey) for the respective bin. (C) Number of positives (red) and negatives (grey) compounds per bin in the percentage histogram

**Figure 6:**
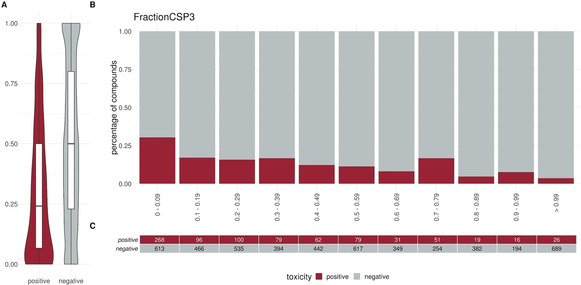
Distribution of the descriptor FractionCSP3 (fraction of sp3 hybridized carbons) between positives and negatives. (A) Box or Violin plots of the distribution of the positives (red) and negatives (grey) compounds in our dataset (without the external test set) (B) Percentage histograms of the dataset. Each bar indicates the percentage of the positives compounds (red) and the percentage of the negatives compounds (grey) for the respective bin. (C) Number of positives (red) and negatives (grey) compounds per bin in the percentage histogram

**Figure 7:**
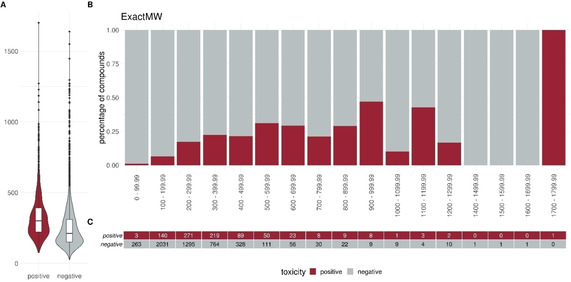
Distribution of the descriptor ExactMW (molecular weight) between positives and negatives. (A) Box or Violin plots of the distribution of the positives (red) and negatives (grey) compounds in our dataset (without the external test set) (B) Percentage histograms of the dataset. Each bar indicates the percentage of the positives compounds (red) and the percentage of the negatives compounds (grey) for the respective bin. (C) Number of positives (red) and negatives (grey) compounds per bin in the percentage histogram.

**Figure 8:**
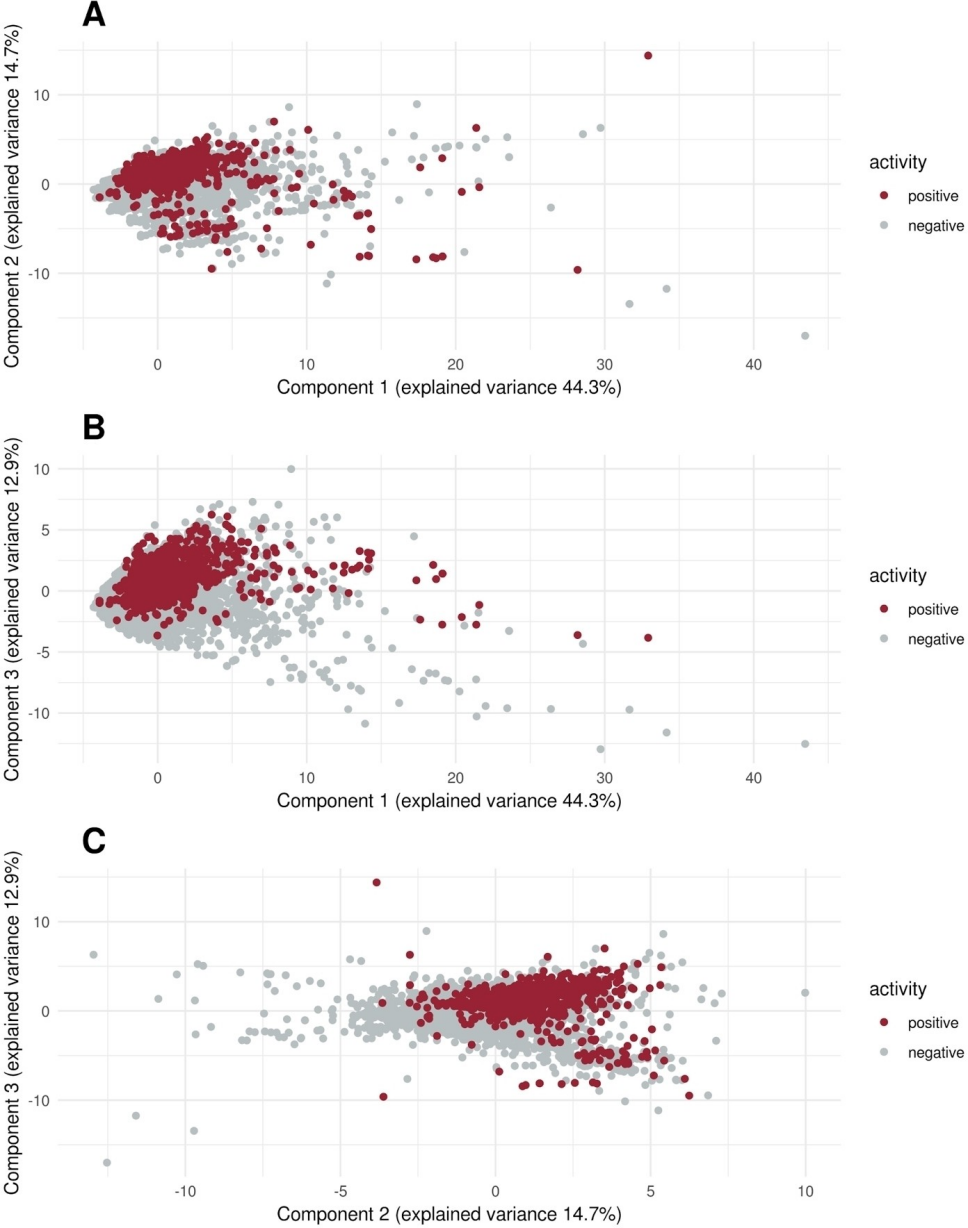
Principal components 1–3. Principal component analysis of the dataset with indication of the positives (red) and negatives (grey). (A) Plot of the first and second principal component (B) Plot of the first and third principal component (C) Plot of the second and third principal component

Subsequently, we analysed the dataset using a PCA to depict the chemical space of the dataset and to see whether the transformation can reveal patterns (see also Figure 8A–C). Although in the PCA no clear separation of the positives and negatives can be seen, it confirmed the observed patterns from the chemical property analysis. The first three principal components explain 71.9 % of the variance. Whereas the first component consists mostly of descriptors related to the size and weight of a molecule, such as “molecular weight”, “LabuteASA” and “number of heavy atoms”, the second component consists of descriptors related to the number of rings, such as the number of aromatic rings or the number of aromatic carbocycles. The third principal component mostly consists of the descriptor “SlogP” with a contribution of over 25 % (see supplement A Figure 1B). This denotes that in the PCA the descriptors which had some distinct characteristics for positive and negative compounds are also highly contributing to components of the PCA and therefore they seem to be important characteristics of the dataset. Analysing the plots of the first and second, first and third and second and third principal components it becomes evident that there are areas in the chemical space that consist only of inactive molecules. Especially for the plot of the first and third and second and third principal component we can see an area which consists mostly of negative compounds (see Figure 1). With the high importance of the SlogP for the third principal component this again highlights the importance of the SlogP. Overall, these results show that, although we did not observe a clear separation, there are areas in the chemical space which only contain negatives and can be distinguished from other areas with positive and negative compounds.

### 3.2 Performance of the Machine Learning Models

For the machine learning models three distinct modelling strategies were used. The first model was a random forest which was built with a smaller subset, containing 1412 compounds of the original dataset, and standard settings for the random forest. This approach was supposed to give a baseline predictivity with a sophisticated descriptor selection but no further model tuning. The second and third model constituted of a traditional machine learning and a deep learning approach. Both approaches used a nested cross validation to choose the model and directly validate the final model on an external validation fold. For the traditional machine learning model, the choice was between four traditional algorithms with different representations and hyperparameters (see section 2.5). For the deep learning model, the representation and algorithm were fixed, but we conducted a hyperparameter search to find the most suitable architecture. Therefore, the two approaches using a nested cross validation were used to investigate (i) if hyperparameter tuning can improve the predictivity of the models, and (ii) whether deep learning is superior to traditional modelling approaches.

Finally, we obtained three models with a very similar balanced accuracy: a random forest with a balanced accuracy of 0.866, a gradient boosting model with a balanced accuracy of 0.894, and a deep learning model with a balanced accuracy of 0.895 (see Table 3). However, the positive predictive performance (PPV) indicates a considerable difference in the models. Whereas the gradient boosting, as well as the deep learning model, have a good PPV of 0.876 and 0.820, respectively, the random forest has a low PPV of 0.295. A low PPV indicates that the model predicts a very high number of false positives. Since all models had differently sized validation datasets, we compiled a test dataset to be used on all models. This allowed us to directly compare the predictivities and further evaluate whether the high PPV of the gradient boosting and deep learning model comes at the expense of a high number of false negatives. Overall, for all models the balanced accuracy dropped more than 12 % when using the external test set. Nevertheless, all models showed a good balanced accuracy of higher than 0.7. The PPV dropped only slightly for the gradient boosting, for the random forest the PPV increased to 0.400, and for the deep learning model it essentially did not change. Nevertheless, validation on the external test set shows that, whereas the gradient boosting and deep learning models predict 50 and 39 compounds as false negatives, the random forest only predicts 20 compounds as false negatives. However, the random forest has a considerable higher number of 189 false positive predictions as compared to 32 and 33 false positives for the gradient boosting and deep learning models respectively. Overall, we observed that hyperparameter tuning can improve the model performance as compared to a baseline model, especially when looking at the false negative and false positive predictions.

### 3.3 Structural Alerts

Since structural alerts are often used to indicate hazards, but also specially to provide a possible mode of action, combining predictive models with structural alerts might lead to a possible starting point for a mechanistic interpretation of a prediction. Therefore, we derived structural alerts by fragmentation of the dataset and implementation of alerts proposed by Nelms et al. and Navel et al.^12^, ^13^ The fragmentation and subsequent selection of the alerts yielded 16 new alerts. The criteria for selection of the alerts was a PPV above 0.6, an occurrence of 9 times or more (which denotes that the alerts are approximately occurring in 1 % of the compounds), and a reasonably specific scaffold. Using the alerts introduced by Navel et al. and Nelms et al. yielded 3 alerts with a PPV above 0.6. Namely, those were the alerts 2‐anilinobenzoic acids (PPV of 0.667), antracene‐9,10‐diones (PPV of 0.844) from Nelms et al. and the alerts aromatic azos (PPV of 0.667) from the paper from Naven et al. However, only the alert antracene‐9,10‐diones was added to the 16 new alerts since, along with the high PPV of 0.884, it was also present more than nine times in the dataset. Overall, we developed 17 highly specific alerts (see Table 2 and supplement C). Since all alerts are very specific, this results in a poor predictivity of the alerts itself (see Table 3). Nevertheless, each alert can be used to indicate hazards with a high specificity. Therefore, if used after the prediction of the models, they can generate insights into the possible mode of action related to the prediction.^24^ In addition, they serve as starting points for read‐across searches, which can complement a similarity search. For this we used our models and the alerts and predicted the mitotoxicity risk for compounds in DrugBank. We then investigated the positive predictions and tried to derive a mode of action.

**Table 2:** Summarized table of identified structural alerts. The occurrence signifies how many molecules were identified in the training dataset using the structural alert. The PPV is the percentage of positive molecules within all molecules which were flagged by the structural alert.

	Structure	SMARTS	Name	Occurrence	PPV
1	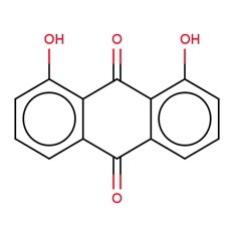	c1cc2 C(=O)c3c(C(=O)c2c(O)c1)c(O)ccc3	Danthron, dihydroanthracene	14	1.000
2	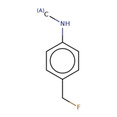	CNc1ccc(C(F))cc1	4‐(fluoromethyl)‐N‐methylaniline	9	0.889
3	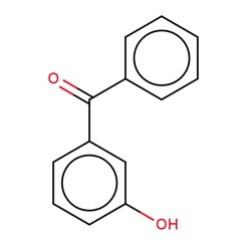	C(=O)(c1cc(O)ccc1)c1ccc(cc1)	P‐hydroxybenzophenone	30	0.867
4	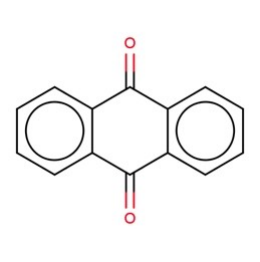	O=C1c2ccccc2 C(=O)c2ccccc21	antracene‐9,10‐diones	32	0.844
5	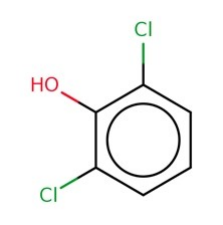	c1(Cl)cccc(c1O)Cl	2,6‐dichlorophenol	12	0.833
6	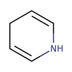	C1=CNC=CC1	dihydropyridine	11	0.818
7	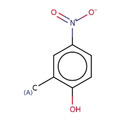	[N+](=O)([O−])c1ccc(c(c1)C)O	2‐methyl‐4‐nitrophenol	11	0.818
8	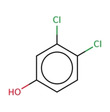	c1(Cl)c(Cl)ccc(c1)O	3,4‐dichlorophenol	10	0.800
9	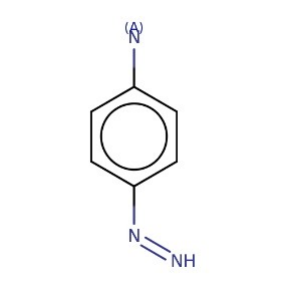	c1(N)ccc(cc1)N=N	4‐diazenylaniline	10	0.800
10	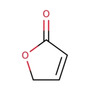	O=C1 C=CCO1	2,5‐dihydrofuran‐2‐one	15	0.733
11	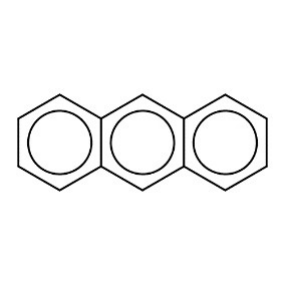	c1ccc2cc3ccccc3cc2c1	anthracene	15	0.733
12	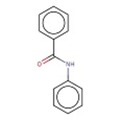	O=C(Nc1ccccc1)c1ccccc1	N‐phenylbenzamide	33	0.697
13	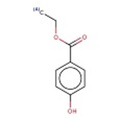	CCOC(=O)c1ccc(O)cc1	ethyl 4‐hydroxybenzoate	23	0.696
14	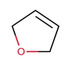	C1=CCOC1	2,5‐dihydrofuran	16	0.688
15	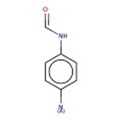	C(=O)(Nc1ccc(N)cc1)	N‐(4‐aminophenyl)formamide	12	0.667
16	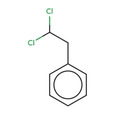	ClC(Cl)Cc1ccccc1	(2,2‐dichloroethyl)benzene	10	0.600
17	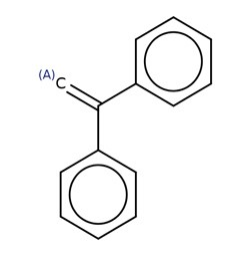	c1c(cccc1)C(=C)c1ccccc1	(1‐phenylethenyl)benzene	32	0.594

**Table 3:** Predictivities of the three trained models and the structural alerts on the training and external Tox21 test dataset. Bold numbers indicate the best performing model according to the metric. The deviations of the deep learning models are due to the generated ensemble of five models.

Model	Random Forest		Gradient Boosting		Deep learning		Structural alerts	
Dataset	Train	Test	Train	Test	Train	Test	Train	Test
Balanced accuracy	0.866	0.743	0.894	0.708	0.895±0.033	**0.760±0.008**	0.598	0.514
Sensitivity	0.942	**0.793**	0.806	0.467	0.820±0.059	0.574±0.018	0.209	0.044
Specificity	0.789	0.692	0.981	**0.948**	0.970±0.007	0.946±0.005	0.987	0.984
PPV	0.295	0.279	0.877	0.573	0.820±0.045	**0.614±0.019**	0.730	0.286
True positives	114	73	533	43	543±51	52±2	173	4
False positives	272	189	75	32	118±27	33±3	64	10
True negatives	1019	425	3926	582	3826±27	580±3	4871	606
False negatives	7	19	128	49	119±39	39±2	654	86

### 3.4 Retrospective Analysis of DrugBank

**Figure 9:**
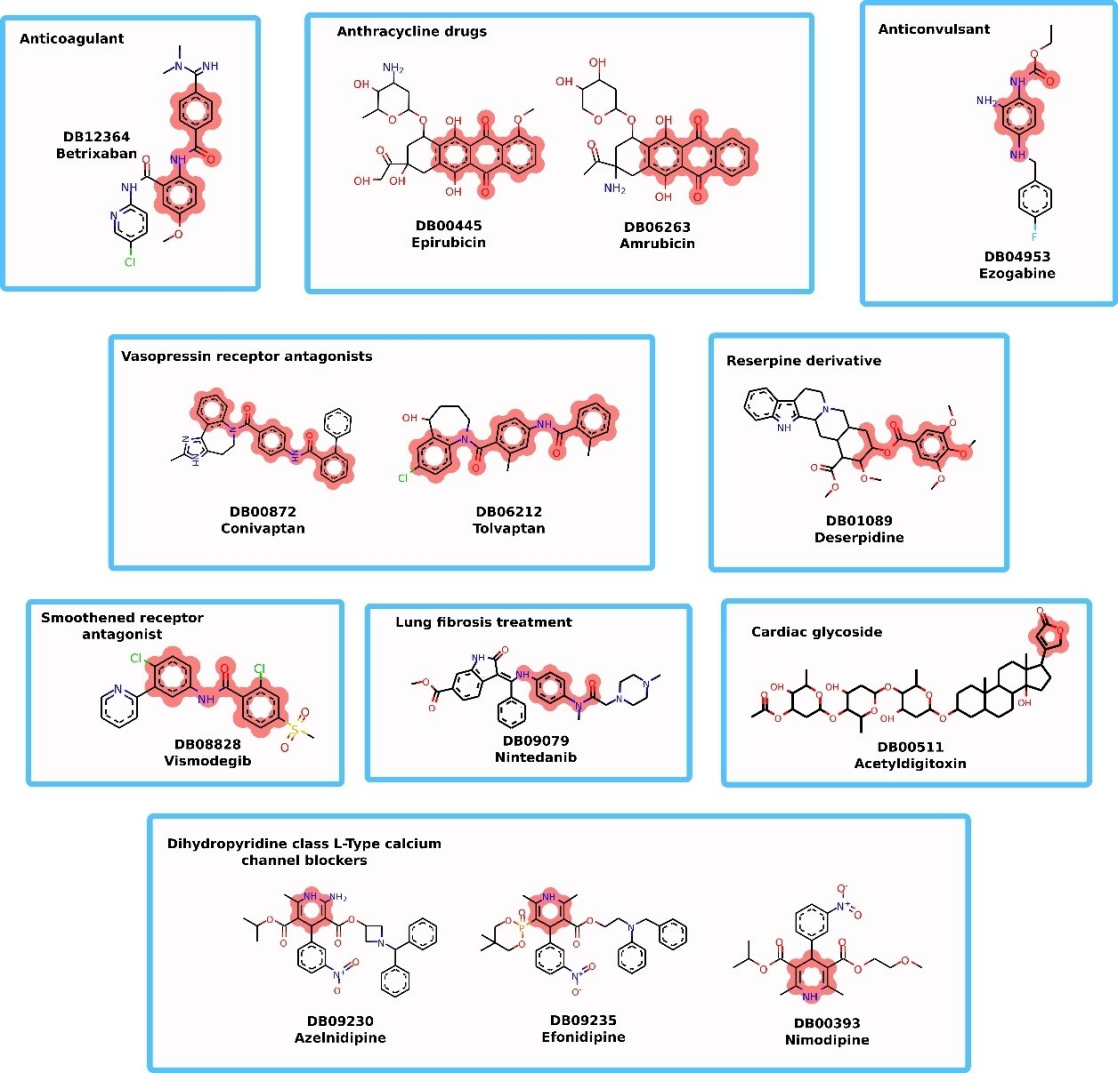
DrugBank positive compounds. All 13 compounds were positive predicted by all three machine learning models and had at least one structural alert.

To demonstrate that structural alerts can help in the understanding of machine learning predictions we used DrugBank for a retrospective analysis. As a first step we predicted all marketed or withdrawn drugs with our models and searched for structural alerts. Subsequently, we investigated all drugs which were predicted as positive by our models. Overall, we had 2278 approved or withdrawn drugs. Out of these 47 were flagged by all models as positive and 52 were flagged by structural alerts. 13 compounds had positive predictions and a structural alert (see Figure 9).

All 47 flagged compounds had a positive SlogP and only acetyldigitoxin, sirolimus and moxidectin had no aromatic cycles. Similarly, out of the 52 molecules with a structural alert all had a positive SlogP and only acetyldigitoxin had no aromatic rings. However, in this case, the aromaticity of compounds is expected, since only the alert 2,5‐dihydrofuran‐2‐one and the alert 2,5‐dihydrofuran do not contain aromatic rings. Subsequently, we examined the available information on the drugs predicted positive by all models. For 9 out of 47 drugs we found evidence for mitochondrial toxicity in the literature (e. g. amrubicin, tolvaptan, atovaquone)^25^, ^26^, ^27^, ^28^, ^29^ (supplement B). An additional 5 drugs had evidence of mitochondrial toxicity, but the literature was not conclusive. For the 52 molecules flagged by structural alerts we found evidence for 6 molecules, for 2 additional molecules the literature was not conclusive. In a case study we tried to explain positive predictions by our models using the structural alert. For this we used the alert anthracenes, which is the alert number 4 and was also reported by Nelms and co‐workers,^13^ therefore we have strong evidence that this substructure is prone to cause mitochondrial toxicity. In our dataset the alert occurred 32 times with a high predictivity of 0.88. Nelms and co‐workers showed a mechanistic explanation which is based on the finding that anthracycline chemotherapeutics cause mitochondrial toxicity through disruption of the ETC and subsequent ROS formation. In DrugBank we found two anthracene derivatives, which were not present in our training dataset. The two anthracyclines were amrubicin and epirubicine, which were flagged by all our trained models and the structural alert. Due to the structural similarity to anthracycline chemotherapeutics we can assume that the mode of action might be similar. In fact, reviewing the available literature we found weak evidence for both compounds to induce mitochondrial toxicity through inhibition of the membrane potential.^30^, ^31^

### 3.5 Discussion

Data mining is an important part for the modelling of toxicities. While small datasets can be analyzed manually, automated and statistical analysis requires larger datasets. These analyses can point towards important structural properties. Our analysis, which revealed the importance of the SlogP for mitochondrial toxicity, is in line with findings from Naven et al., who experimentally found a correlation between the logP and uncoupling of oxidative phosphorylation.^12^ In addition, we found a link between the size of the molecules and their mitochondrial toxicity. Along with the SlogP this could be an indicator that the drugs interact with specific proteins or transporters which require certain properties like a specific size or hydrophobicity. For example, it was shown that rotenone and several azoles induce mitochondrial toxicity through inhibition of the electron transport chain.^32^, ^33^ Recently, it was shown that amoxicillin/clanvulanate and ciprofloxacin induce mitochondrial toxicity via the opening of the mitochondrial membrane permeability transition pore located in the outer membrane.^34^ Both findings highlight the association of the inhibition of certain mitochondrial proteins with mitochondrial toxicity. In addition, to elucidate these mechanisms the compounds have to cross the cell membrane and might even have to cross the outer mitochondrial membrane, underlining the hypothesis that mitotoxic compounds require certain properties which allow them to bind to specific (off‐)targets. Therefore, large datasets can help to elucidate molecular properties related to toxicities and thus also indirectly related to the binding of their target.

Further, large datasets can be used to train machine learning models to predict hazards in very early stages of the drug discovery process. We showed that the deep learning model as well as the gradient boosting model had a good performance on the training and test set. In addition, we observed that the random forest had a worse performance with respect to the identification of positives. Whereas the other two models could identify the positives with a PPV >0.8, the random forest had a PPV of 0.3 and thus predicted many false positives. This highlights that the modelling process is important and that a grid search over different algorithms or architectures can increase the predictivity. However, an external test set should always be used after the final model is trained for a thorough evaluation. It was unexpected to see a drop in the sensitivity by 0.33 and 0.24 for the gradient boosting and deep learning model, respectively, since both are validated externally during the nested cross validation. Therefore, this should prevent large differences in the predictivity between training and test dataset. The large differences in the predictivity between training and test dataset could be due to an internal bias of the dataset, which would imply that the dataset contains very similar molecules. Therefore, the validation data of the inner cross‐validation is very similar to the training data, whereas the test dataset differs substantially from the training and validation data. However, using the local outlier factor algorithm^22^ for applicability domain estimation, the predictivity of the models did not increase when the outliers were removed. It has to be noted that the external test set was a dataset obtained for the inhibition of mitochondrial membrane potential. Therefore, the endpoint from the high throughput screening could also be responsible for the decrease in performance, since data from such screenings can be very noisy. Finally, the deep learning model yielded no advantage over traditional machine learning approaches, however, we only trained simple feed forward networks, thus more sophisticated architectures could yield a better performance.

Although modelling is a very important part of *in silico* toxicology, to be acceptable, also for regulatory purposes, models have to have “a mechanistic interpretation, if possible” (according to OECD Guideline on Validation of (Q)SAR Models).^35^ Therefore, in recent years, criticism on machine learning as a black box became more pronounced.

Nevertheless, to overcome potential hazards or evaluate a compound, predictive models are a fast and cheap means to decide on an appropriate strategy to proceed with e. g. further development of such a compound. Therefore, structural alerts could be used to indicate potential mechanisms of positive predicted compounds, thus helping to explain predictions of black box models. In these cases, structural alerts can be beneficial to gain insight into potential mode of actions. While machine learning models can be good estimators to predict new data, structural alerts, as seen in our results, are not predictive by themselves. In addition, not all alerts are predictive for other datasets. For our dataset only two of the alerts reported by Nelms et al. showed a clear predictivity for activity, while others were more likely to flag inactive molecules. This could be due to their small dataset size, which might hinder the generalization. Yet it could also be due to noise in our dataset containing high throughput screening data, which is known to be of lower quality than curated and experimentally tested data. Hence, alerts should not be used as a predictive model, but rather as an indicator for potential further investigations in a read across approach as also suggested by Alves et al.^36^ Importantly, they do not provide a similarity based starting point as is commonly used in read‐across, but the alert can lead to a more diverse set of mechanistically related compounds. Conclusively, combining data analysis with machine learning and structural alerts can lead to novel insights into toxicological endpoints. This approach can help in the drug development pipeline to identify hazards early on, gain a better mechanistic understanding of the underlying mode of action and propose valuable structural modifications.

## 4 Conclusions

With our approach we compiled the biggest reported dataset on mitochondrial toxicity so far. By data analysis we could observe trends for important properties, with the most promising being SlogP. In addition, we could show that the dataset is suitable for training of machine learning models. We showed that structural alerts by themselves do not exhibit good predictivity when used for the prediction of a dataset. Nevertheless, we could show that the combination of machine learning with structural alerts can be a powerful tool to predict hazards and derive mechanistic insight.

## Data and Code Availability

The dataset and the corresponding models can be found in our GitHub repository at https://github.com/PharminfoVienna/Mitochondrial‐Toxicity.

## Conflict of Interest

None declared.

## Supporting information

As a service to our authors and readers, this journal provides supporting information supplied by the authors. Such materials are peer reviewed and may be re‐organized for online delivery, but are not copy‐edited or typeset. Technical support issues arising from supporting information (other than missing files) should be addressed to the authors.

Supplementary
