# Supplementary material for: Using Machine Learning Methods and Structural Alerts for Prediction of Mitochondrial Toxicity
Source: Mol Inform. 2020 Mar 23;39(5):2000005. doi: 10.1002/minf.202000005 (PMC7317375; doi:10.1002/minf.202000005)
Supplement: Supplementary file 1 — Supplementary [file MINF-39-2000005-s001.pdf]

molecular informatics

Supporting Information

Using Machine Learning Methods and Structural Alerts for Prediction of Mitochondrial Toxicity

Jennifer Hemmerich[+], Florentina Troger[+], Barbara Füzi, and Gerhard F.Ecker*© 2020 The Authors. Published by Wiley-VCH Verlag GmbH & Co. KGaA. This is an open access article under the terms of the Creative Commons Attribution License, which permits use, distribution and reproduction in any medium, provided the original work is properly cited. The copyright line for this article was changed on May 13, 2020 after original online publication.

The supplemental tables B and C as well as the model information and the used datasets are also available as *.xls / *.sdf files in our github repository at:

https://github.com/PharminfoVienna/Mitochondrial-Toxicity

SUPPLEMENT A

Table A List of RDKit descriptors used for the dataset analysis

SlogP	Smarts LogP, Octanol Water Partition Coefficient
LabuteASA	Labute's Approximate Surface Area, approximated surface area of a molecule (Labute, 2000)
TPSA	Total Polar surface area
ExactMW	Molecular weight
NumRotatableBonds	Number of rotatable bonds
NumHBD	Number of hydrogen bond donors
NumHBA	Number of hydrogen bond acceptors
NumAmideBonds	Number of amide bonds
NumHeteroAtoms	Number of hetero atoms
NumHeavyAtoms	Number of heavy atoms
NumAtoms	Number of atoms
NumRings	Number of rings
NumAromaticRings	Number of aromatic rings
NumSaturatedRings	Number of saturated rings
NumAliphaticRings	Number of aliphatic rings
NumAromaticHeterocycles	Number of aromatic heterocycles
NumSaturatedHeterocycles	Number of saturated heterocycles
NumAliphaticHeterocycles	Number of aliphatic heterocycles
NumAromaticCarbocycles	Number of aromatic carbocycles

NumSaturatedCarbocycles	Number of saturated carbocycles
NumAliphaticCarbocycles	Number of aliphatic carbocycles
FractionCSP3	Fraction of sp3 hybridized Carbons
SMR	Molecular refractivity

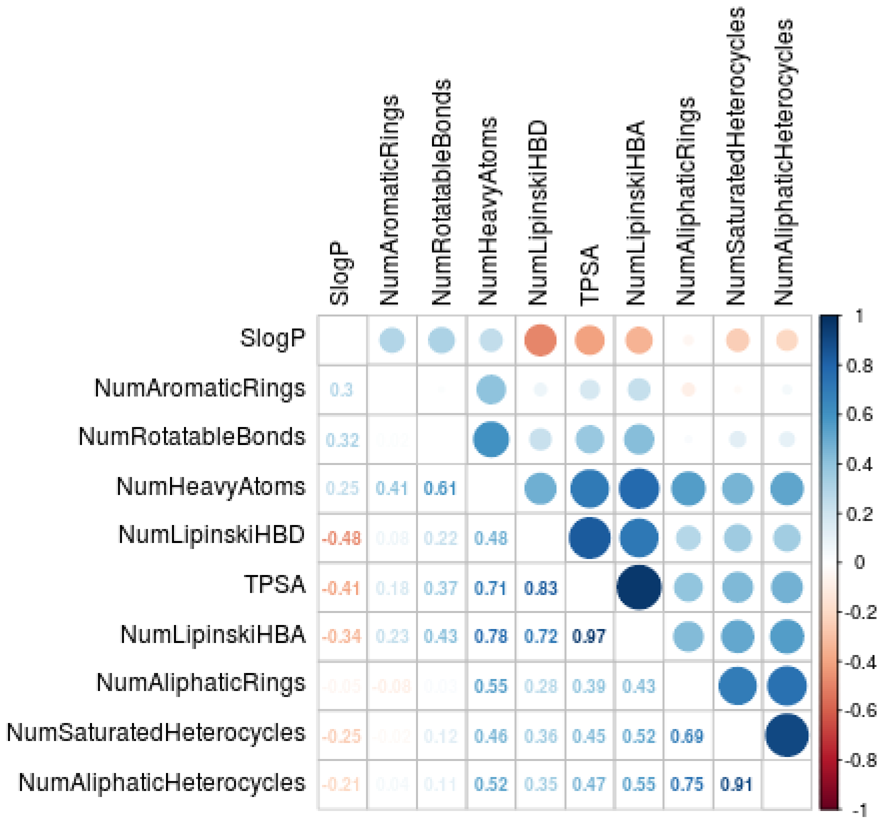

Figure A Correlation matrix of the descriptors used for the random forest

The size and color of the dots in the upper triangle represents the correlation of the respective. Larger dots indicate a higher contribution. The lower triangle represents the correlation as a numeric vale.

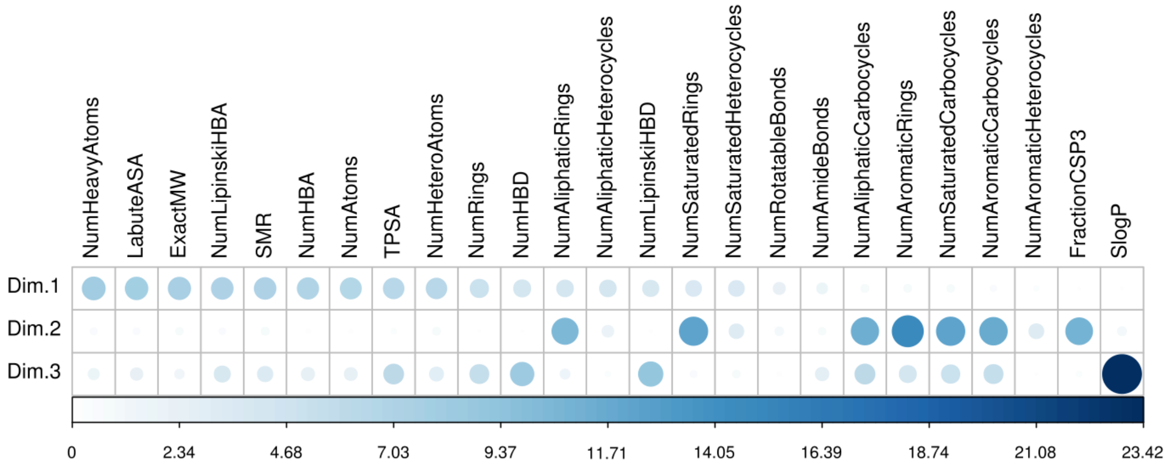

Figure B PCA contribution of variables.
The size and color of the dots represents the contribution of the respective descriptor to the principal component. Larger dots indicate a higher contribution.

Supplement B

Columns:
DrugBankID: Identifier of the DrugBank database
Name: Generic name of the drug as defined in DrugBank
Evidence: Whether literature was found to support the hypothesis that the compound is mitotoxic, “no literature found” indicates that, with the used search terms, we did not get any search results
Paper: Literature supporting the evidence (Please note this is NOT an exhaustive list, merely examples supporting the assumption of a toxicity mechanism involving mitochondria)
Contains structural alert: Whether the molecule was flagged by one of our 17 structural alerts
Positive predicted: Whether the compound was predicted as positive by all our machine learning models

DBID	Name	Evidence	Paper	Contains Structural Alert	Positive Predicted
DB09061	Cannabidiol	yes	Finsterer et al. doi: 10.1016/j.eplepsyres.2017.07.003<br>Singh et al. doi: 10.1007/s12031-015-0545-2.		yes
DB08896	Regorafenib	yes	Weng et al. doi: 10.1016/j.tox.2014.11.002.		yes
DB06263	Amrubicin	yes	Hanada et al. doi: 10.1111/j.1349-7006.2006.00318.x	yes	yes
DB06212	Tolvaptan	yes	Mosedale et al. doi: 10.1093/toxsci/kfx206.<br>Woodhead et al. doi: 10.1093/toxsci/kfw193.	yes	yes
DB04823	Oxyphenisatin	yes	Morrison et al. doi: 10.1002/cam4.107.		yes
DB01117	Atovaquone	yes	Pinho et al. 10.1111/bph.12186<br>Oliveira et al. doi: 10.1007/s13318-015-0294-1.		yes
DB01076	Atorvastatin	yes	Broniarek et al. doi: 10.1016/j.abb.2017.12.003<br>Sadighara et al. doi: 10.1111/bcpt.12656<br>Pal et al. doi: 10.1016/j.fct.2015.05.016.		yes
DB00445	Epirubicin	yes	Huang et al. doi: 10.1007/s10495-018-1450-2<br>Yoshida et al. doi: 10.1111/cas.12567<br>Guven et al. doi: 10.1016/j.acthis.2006.09.007	yes	yes
DB00255	Diethylstilbestrol	yes	Berson et al. doi: 10.1016/S0016-5085(98)70590-6		yes
DB01623	Thiothixene	yes	Burkhardt et al. doi: 10.1002/ana.410330516	yes	
DB08880	Teriflunomide	yes	Xuan et al. doi: 10.1016/j.tox.2018.02.003.	yes	
DB08901	Ponatinib	yes	Paech et al. doi: 10.1016/j.tox.2018.01.005<br>Mingard et al. doi: 10.1002/jat.3551.	yes	
DB12792	Boscalid	inconclusive	Regueiro et al. doi: 10.1016/j.envres.2015.03.013.		yes
DB06210	Eltrombopag	inconclusive	Kalota et al. doi: 10.1371/journal.pone.0126691.		yes
DB05109	Trabectedin	inconclusive	Gajate et al. doi: 10.1074/jbc.M204644200<br>Atmaca et al. doi: 10.1016/j.toxlet.2013.06.213.		yes
DB00570	Vinblastine	inconclusive	Madhu et al. doi: 10.1002/jez.1917.		yes
DB00539	Toremifene	inconclusive	Kallio et al. doi: 10.1007/s10495-005-2137-z	yes	
DB01094	Hesperetin	contradicting	Wu et al. doi: 10.1007/s13277-015-4176-6<br>Shagirtha et al. doi: 10.1177/0748233716665301		yes
DB00540	Nortriptyline	contradicting	Yuan et al. doi: 10.1016/j.ejphar.2015.06.007<br>Zhang et al. doi: 10.1161/STROKEAHA.107.496810.	yes	
DB14011	Nabiximols	no			yes
DB13164	Olmutinib	no			yes
DB12070	Letermovir	no			yes
DB11828	Neratinib	no			yes
DB11431	Moxidectin	no			yes
DB09235	Efonidipine	no		yes	yes
DB09230	Azelnidipine	no		yes	yes
DB09079	Nintedanib	no		yes	yes
DB09048	Netupitant	no			yes
DB08976	Floctafenine	no			yes
DB08906	Fluticasone furoate	no			yes
DB08899	Enzalutamide	no			yes
DB08864	Rilpivirine	no			yes
DB08828	Vismodegib	no		yes	yes
DB08827	Lomitapide	no			yes
DB08820	Ivacaftor	no			yes
DB06414	Etravirine	no			yes
DB06249	Arzoxifene	no			yes
DB04953	Ezogabine	no		yes	yes
DB01419	Antrafenine	no			yes
DB01201	Rifapentine	no			yes
DB01180	Rescinnamine	no			yes
DB01089	Deserpidine	no		yes	yes
DB00393	Nimodipine	no		yes	yes
DB00157	NADH	no		yes	
DB00381	Amlodipine	no		yes	
DB00434	Cyproheptadine	no		yes	
DB00768	Olopatadine	no		yes	
DB00793	Haloprogin	no		yes	
DB00875	Flupentixol	no		yes	
DB00882	Clomifene	no		yes	
DB00924	Cyclobenzaprine	no		yes	
DB01023	Felodipine	no		yes	
DB01068	Clonazepam	no		yes	
DB01142	Doxepin	no		yes	
DB01239	Chlorprothixene	no		yes	
DB01411	Pranlukast	no		yes	
DB01544	Flunitrazepam	no		yes	
DB01624	Zuclopenthixol	no		yes	
DB04868	Nilotinib	no		yes	
DB04920	Clevidipine	no		yes	
DB04938	Ospemifene	no		yes	
DB04948	Lofexidine	no		yes	
DB06228	Rivaroxaban	no		yes	
DB06605	Apixaban	no		yes	
DB06708	Lumefantrine	no		yes	
DB06712	Nilvadipine	no		yes	
DB08810	Cinitapride	no		yes	
DB08968	Fominoben	no		yes	
DB09089	Trimebutine	no		yes	
DB09136	Isosulfan blue	no		yes	
DB09167	Dosulepin	no		yes	
DB09236	Lacidipine	no		yes	
DB11526	Masitinib	no		yes	
DB13967	Patent Blue	no		yes	
DB12364	Betrixaban	no literature found		yes	yes
DB09219	Bisoxatin	no literature found			yes
DB09151	Flutemetamol (18F)	no literature found			yes
DB00890	Dienestrol	no literature found			yes
DB00877	Sirolimus	no literature found			yes
DB00872	Conivaptan	no literature found		yes	yes
DB00693	Fluorescein	no literature found			yes
DB00511	Acetyldigitoxin	no literature found		yes	yes
DB09237	Levamlodipine	no literature found		yes	
DB13114	Amitriptylinoxide	no literature found		yes	
DB13711	Tritoqualine	no literature found		yes	

Supplement C

	SMARTS	Name	Occurrence	PPV
1	c1cc2C(=O)c3c(C(=O)c2c(O)c1)c(O)ccc3	Danthron, dihydroanthracene	14	1,000
2	CNc1ccc(C(F))cc1	4-(fluoromethyl)-N-methylaniline	9	0.889
3	C(=O)(c1cc(O)ccc1)c1ccc(cc1)	P-hydroxybenzophenone	30	0.867
4	O=C1c2ccccc2C(=O)c2ccccc21	antracene-9,10-diones	32	0.844
5	c1(Cl)cccc(c1O)Cl	2,6-dichlorophenol	12	0.833
6	C1=CNC=CC1	dihydropyridine	11	0.818
7	[N+](=O)([O-])c1ccc(c(c1)C)O	2-methyl-4-nitrophenol	11	0.818
8	c1(Cl)c(Cl)ccc(c1)O	3,4-dichlorophenol	10	0.800
9	c1(N)ccc(cc1)N=N	4-diazenylaniline	10	0.800
10	O=C1C=CCO1	2,5-dihydrofuran-2-one	15	0.733
11	c1ccc2cc3ccccc3cc2c1	anthracene	15	0.733
12	O=C(Nc1ccccc1)c1ccccc1	N-phenylbenzamide	33	0.697
13	CCOC(=O)c1ccc(O)cc1	ethyl 4-hydroxybenzoate	23	0.696
14	C1=CCOC1	2,5-dihydrofuran	16	0.688
15	C(=O)(Nc1ccc(N)cc1)	N-(4-aminophenyl)formamide	12	0.667
16	ClC(Cl)Cc1ccccc1	(2,2-dichloroethyl)benzene	10	0.600
17	c1c(cccc1)C(=C)c1ccccc1	(1-phenylethenyl)benzene	32	0.594
